# TypiCal but DeliCate Ca^++^re: Dissecting the Essence of Calcium Signaling Network as a Robust Response Coordinator of Versatile Abiotic and Biotic Stimuli in Plants

**DOI:** 10.3389/fpls.2021.752246

**Published:** 2021-11-25

**Authors:** Neelesh Patra, Shruthi Hariharan, Hena Gain, Mrinal K. Maiti, Arpita Das, Joydeep Banerjee

**Affiliations:** ^1^Department of Biotechnology, Indian Institute of Technology Kharagpur, Kharagpur, India; ^2^Agricultural and Food Engineering Department, Indian Institute of Technology Kharagpur, Kharagpur, India; ^3^Department of Genetics and Plant Breeding, Bidhan Chandra Krishi Viswavidyalaya, Mohanpur, India

**Keywords:** calcium, calmodulin, genetic intervention, signaling, stress

## Abstract

Plant growth, development, and ultimately crop productivity are largely impacted by the interaction of plants with different abiotic and biotic factors throughout their life cycle. Perception of different abiotic stresses, such as salt, cold, drought, heat, and heavy metals, and interaction with beneficial and harmful biotic agents by plants lead to transient, sustained, or oscillatory changes of [calcium ion, Ca^2+^]_cyt_ within the cell. Significant progress has been made in the decoding of Ca^2+^ signatures into downstream responses to modulate differential developmental and physiological responses in the whole plant. Ca^2+^ sensor proteins, mainly calmodulins (CaMs), calmodulin-like proteins (CMLs), and others, such as Ca^2+^-dependent protein kinases (CDPKs), calcineurin B-like proteins (CBLs), and calmodulin-binding transcription activators (CAMTAs) have played critical roles in coupling the specific stress stimulus with an appropriate response. This review summarizes the current understanding of the Ca^2+^ influx and efflux system in plant cells and various Ca^2+^ binding protein-mediated signal transduction pathways that are delicately orchestrated to mitigate abiotic and biotic stresses. The probable interactions of different components of Ca^2+^ sensor relays and Ca^2+^ sensor responders in response to various external stimuli have been described diagrammatically focusing on established pathways and latest developments. Present comprehensive insight into key components of the Ca^2+^ signaling toolkit in plants can provide an innovative framework for biotechnological manipulations toward crop improvability in near future.

## Introduction

Calcium ion (Ca^2+^) acts as a critically important relay hub for many biological messages. Spatio-temporal changes in Ca^2+^ concentration inside cellular compartments and the whole cell itself have an important function in the signal transduction networks of all eukaryotes. The presence of Ca^2+^ pumps in the most primitive bacteria suggests that from the very beginning of life, sensing and transporting of Ca^2+^ have emerged as an absolute homeostatic necessity for cells to combat cytotoxicity (Verkhratsky and Parpura, 2014). Due to its sessile nature, plants have developed sophisticated adaptation mechanisms for counteracting environmental cues, such as abiotic and biotic factors (Kissoudis et al., 2014). In normal conditions, plant cells maintain a relatively low concentration of free cytosolic calcium ([Ca^2+^]_cyt_), of around 100 nM compared to extracellular surroundings and intracellular storage compartments where Ca^2+^ concentration ranges in the mM scale (Trewavas and Malhó, 1998). [Ca^2+^]_cyt_ level is strictly regulated at the cellular level and tiny fluctuations in [Ca^2+^]_cyt_ can be decoded as useful external inklings for activation of downstream signaling components that include various proteins.

Upon sensing aggravation, the ubiquitous change that happens within the plant system is the shift in free [Ca^2+^]_cyt_ which is raised either from the external medium or from the intracellular/sub-cellular compartments having higher Ca^2+^ concentration compared to the [Ca^2+^]_cyt_ (Srivastava et al., 2013). Based on electrophysiological properties, there are three distinct Ca^2+^ transporting channels, categorized as hyperpolarization-activated calcium channels (HACCs), depolarization-activated calcium channels (DACCs), and voltage-independent calcium channels (VICCs) (Swarbreck et al., 2013). Non-selective cation channels (NSCCs) comprised of depolarization-activated NSCCs (DA-NSCCs), voltage-independent NSCCs (VI-NSCCs), and hyperpolarization-activated NSCCs (HA-NSCCs) are the ubiquitous group of ion channels that do not discriminate between essential and toxic cations of either monovalent or divalent nature and are found in the plasma membrane, tonoplast, and another endomembrane of plant tissues (Demidchik and Maathuis, 2007). [Ca^2+^]_cyt_ elevation is mainly a result of the Ca^2+^ influx through membrane-localized Ca^2+^ accessible ion channels, such as cyclic nucleotide-gated channels (CNGCs), ionotropic glutamate receptors (GLRs), two-pore channel 1 (TPC1), annexins, mechanosensitive-like channels (MSLs), mid1-complementing activity channels (MCA), and hyperosmolarity-induced [Ca^2+^]_cyt_ increase channels (OSCAs) (Kudla et al., 2018). Along with the plasma membrane, vacuole, the largest intracellular Ca^2+^ reservoir, is imperative for determining the concentration of [Ca^2+^]_cyt_ in plants. Several channels have been detected in the vacuolar tonoplast to be involved in Ca^2+^ influx in the cytosol, such as voltage-dependent slow vacuolar/TPC1 (SV/TPC1) and CNGCs. Moreover, the channels for inositol-1,4,5-trisphosphate (IP3) and cyclic-ADP ribose (cADPR) are expected to be involved in determining the concentration of [Ca^2+^]_cyt_ in plants, but the molecular elucidation is not yet clear (Schönknecht, 2013). On the other hand, efflux in a cell is mostly controlled by two types of active Ca^2+^ transport systems, namely, P-type Ca^2+^-ATPase (Huda et al., 2013) and cation exchanger (CAX) family Ca^2+^:H^+^ exchangers (Schönknecht, 2013; Pittman and Hirschi, 2016).

Certain external stimuli mediate specific transient boosts in [Ca^2+^]_cyt_ along with stimulus-dependent amplitude and shape (e.g., sinusoidal and square-wave) in a temporal manner (Qudeimat and Frank, 2009). These transient boosts in [Ca^2+^]_cyt_, known as Ca^2+^ signatures, depend on Ca^2+^ channels available in cell membranes or in the sub-cellular compartments within the cell along with the network of Ca^2+^ transporting proteins (Qudeimat and Frank, 2009). These Ca^2+^ signatures are eventually sensed, decoded, and transmitted to the downstream signaling components. Plant Ca^2+^ sensor proteins are classified into two groups, namely, sensor relays and sensor responders. Plant proteins, namely, calmodulins (CaMs), calmodulin-like proteins (CMLs), and calcineurin B-like proteins (CBLs) are the components of sensor relays that do not possess any enzymatic functions but they possess one or more E and F helices (EF) hands for binding with Ca^2+^ ions and subsequent modulation in their protein conformation (Meena et al., 2019). Contrarily, sensor responders, such as calcium-dependent protein kinases (CDPKs or CPKs) and CBL-interacting protein kinases (CIPKs), possess enzymatic functions for interacting with specific downstream proteins through phosphorylation and dephosphorylation (Hashimoto and Kudla, 2011).

Plant genomes maintain a diverse array of Ca^2+^ signaling components in different plant species. *Arabidopsis* genome contains at least 7 *CaM*, 50 *CML*, 10 *CBL*, and 34 *CDPK* genes (Zeng et al., 2017). Ca^2+^ ion and CaM can interact and regulate the activity of many transcription factors (TFs) in the plant and among the reported TFs, calmodulin-binding transcription activators (CAMTAs) are the largest well-characterized CaM-binding TF in all multicellular organisms, more or less conserved from plants to human (Finkler et al., 2007). Ca^2+^/CaMs binding regulates transcriptional activity of CAMTAs on several downstream genes involved in abiotic stress tolerance and disease resistance (Du et al., 2009).

In this review, we have attempted to highlight the role of Ca^2+^ ion and associated signaling armor viz., CaM, CMLs, CDPKs, CBLs, and CAMTAs, toward harmonizing plant responses to mitigate various abiotic and biotic stresses.

## Ca^2+^ Signatures in Plant-Abiotic Factors Interaction

Taking lessons of the escalating environmental vagaries, attempts have been made to underpin the impact of different Ca^2+^ signatures and their downstream components toward elevating abiotic stress tolerance in plants.

### Salt Stress

Salt stress creates an unfavorable environment for plant growth through osmotic and ionic stresses. Higher concentrations of soluble ions in the rhizosphere reduce the soil water potential hindering water uptake by plant roots concurrently creating osmotic stress or dehydration stress. Eventually, the gradual accumulation of salt ions inside the plant tissue causes ionic stress to the plant. The salt overlay sensitive (SOS) pathway is a well-established Ca^2+^-dependent signaling cascade for Na^+^ ion homeostasis. Na^+^ ion probably enters into the cell through anatomical leaks or embroiling hyperpolarization-activated NSCC to eventuate a rapid spike in the calcium-gated channel (Ji et al., 2013). The spike in the Ca^2+^-gated channel activates calcium-binding protein (CBP) SOS3 through myristoylation, a post-translational modification of SOS3, to mediate subsequent binding and activation of a serine/threonine kinase protein, SOS2 (Mahajan et al., 2008). Finally, the SOS2-SOS3 complex activates SOS1 available in the cell membrane to remove excess Na^+^ ions from the cell (Mahajan et al., 2008). In *Arabidopsis*, calcineurin B-like 4 (CBL4) acts as a SOS3 protein and CBL-interacting protein kinase 24 (CIPK24), a serine/threonine protein kinase, performs as SOS2 (Yang et al., 2019). Further, the study identified a SOS3-like calcium-binding protein 8 (SCaBP8), later named calcineurin B-like 10 (CBL10), that can bind with the SOS2 protein and intercedes phosphorylation for subsequent downstream signaling (Quan et al., 2007; Lin et al., 2009). CBL4 is mostly functional in root tissue, while CBL10 is mostly linked with the shoot and critically associated with the reproductive tissue development of different plants and the signaling of both CBL4 and CBL10 under salt stress work independently (Yang et al., 2019). CBL10 directly acts on translocon of the outer membrane of chloroplasts 34 (TOC34) protein and reduces the GTPase activity of TOC34 (Cho et al., 2016). For TOC34 protein translocation in the plastid, GTP binding and hydrolysis are highly essential while transportation of several proteins inside the chloroplast is mediated by Ca^2+^/CaM signaling (Chigri et al., 2005). Since CBL10 and TOC34 are expressed in different types of plastids in plants (Cho et al., 2016), it can be speculated that salt stress signaling modulates the functioning of various proteins in the cytoplasm and other organelles. Moreover, another finding reveals that the Ca^2+^ dynamics in the cytosol and nucleus are differentially regulated to control overall stress signaling in *Arabidopsis* (Huang et al., 2017). CBL10 also interacts with CIPK24 in addition to various other CIPKs and activates several transporters in vacuoles like H^+^/Ca^2+^ antiporter (also known as CAX1), H^+^-ATPase transporter, and Na^+^/H^+^ transporter (NHX1) in *Arabidopsis* (Yang et al., 2019) for maintaining Na^+^ and K^+^ ion homeostasis in the plant cell. Besides, Annexin1 has been found to regulate Ca^2+^ influx in the root epidermal tissue of *Arabidopsis* upon sensing salt stress (Laohavisit et al., 2013).

Another crosstalk has been detected in *Arabidopsis* and other plants to combat salt stress through phosphatidic acid (PA)-mediated signaling cascade (Yu et al., 2010; McLoughlin et al., 2012). Some membrane phospholipids, namely, phosphatidylcholine and phosphatidylethanolamine, are hydrolyzed by phospholipase D (PLD) to produce PA and the residual head group. Moreover, the hydrolysis of phosphatidylinositol lipid is carried out by phospholipase C to produce diacylglycerol (DAG) that is eventually phosphorylated by DAG kinase to produce PA (Yao and Xue, 2018). Various stresses animate transient increase in PA which eventually bind with mitogen-activated protein kinase 6 (MPK6) to trigger phosphorylation of SOS1 protein, facilitating Na^+^ extrusion from the cell and other TFs, having a direct effect on salt-stress alleviation (Yu et al., 2010). Additionally, PA binds with two sucrose non-fermenting-1 (SNF1)-related protein kinase 2 (SnRK2.4 and SnRK2.10) proteins for shaping root architecture under salt stress (McLoughlin et al., 2012; Julkowska et al., 2015). A comprehensive study unravels that the binding of mitogen-activated protein kinase kinase 7 (MKK7) and MKK9 with PA is crucial for their translocation to the membrane. These interactions trigger MPK6 to activate SOS1 in the membrane (Shen et al., 2019).

**Figure 1:**
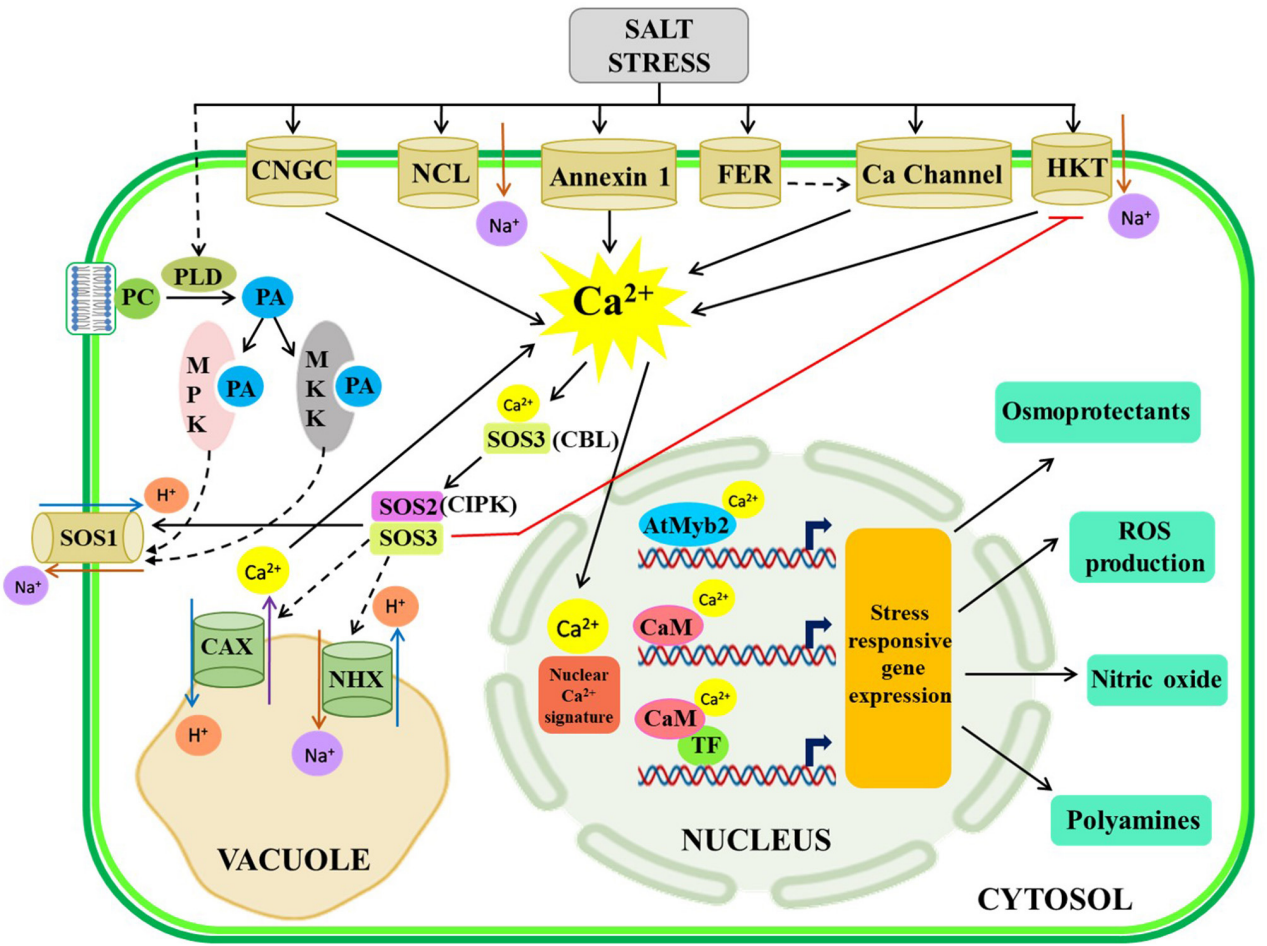
Illustrative representation of crosstalk among Ca^2+^ signaling components under the salt-stressed condition. During the salt-stressed condition, Na+ is transported into the cytoplasm through different plasma membrane-localized channels leading to a spike in [Ca^2+^]_cyt_, which activates SOS pathway and PA-mediated signaling, necessary for extrusion of Na+ from the cytosol to the vacuole and extracellular space and subsequent stress-responsive gene expression for production of osmoprotectants, ROS, nitric oxide, and polyamines. The solid arrow represents direct activation; the dotted arrow represents indirect activation via some intermediates. NCL-Na^+^/Ca^2+^exchanger like protein, HKT, high-affinity K+ transport, FER-FERONIA, PCphosphatidylcholine; SOS, salt overlay sensitive; ROS, reactive oxygen species.

The nuclear Ca^2+^ signature is mediated in plants by CaM and CDPK which are basically EF-hand motif-containing Ca^2+^ sensors (Kim et al., 2009). In response to salt stress in common ice plants (*Mesembryanthemum crystallinum*), a CDPK (McCDPK1) has been isolated containing a nuclear localization signal (NLS) for translocation of McCDPK1 into the nucleus to mitigate salt stress (Patharkar and Cushman, 2000). Several Ca^2+^ binding TFs viz., AtNIG1 (*Arabidopsis thaliana* NaCl-inducible gene 1), have been detected for decoding of Ca^2+^ signaling in both cytosol and the nucleus (Kim and Kim, 2006), which is an integral part of the promoter region of various salinity stress-responsive genes (Chinnusamy et al., 2003). CaM isoforms have also been discovered in soybeans that modulated the salt-induced Ca^2+^ signaling by activating an MYB-type TF, which is an upstream regulator of several salt and dehydration responsive genes (Yoo et al., 2005). The overall Ca^2+^ signaling under salt stress is depicted in Figure 1.

### Drought Stress

**Figure 2:**
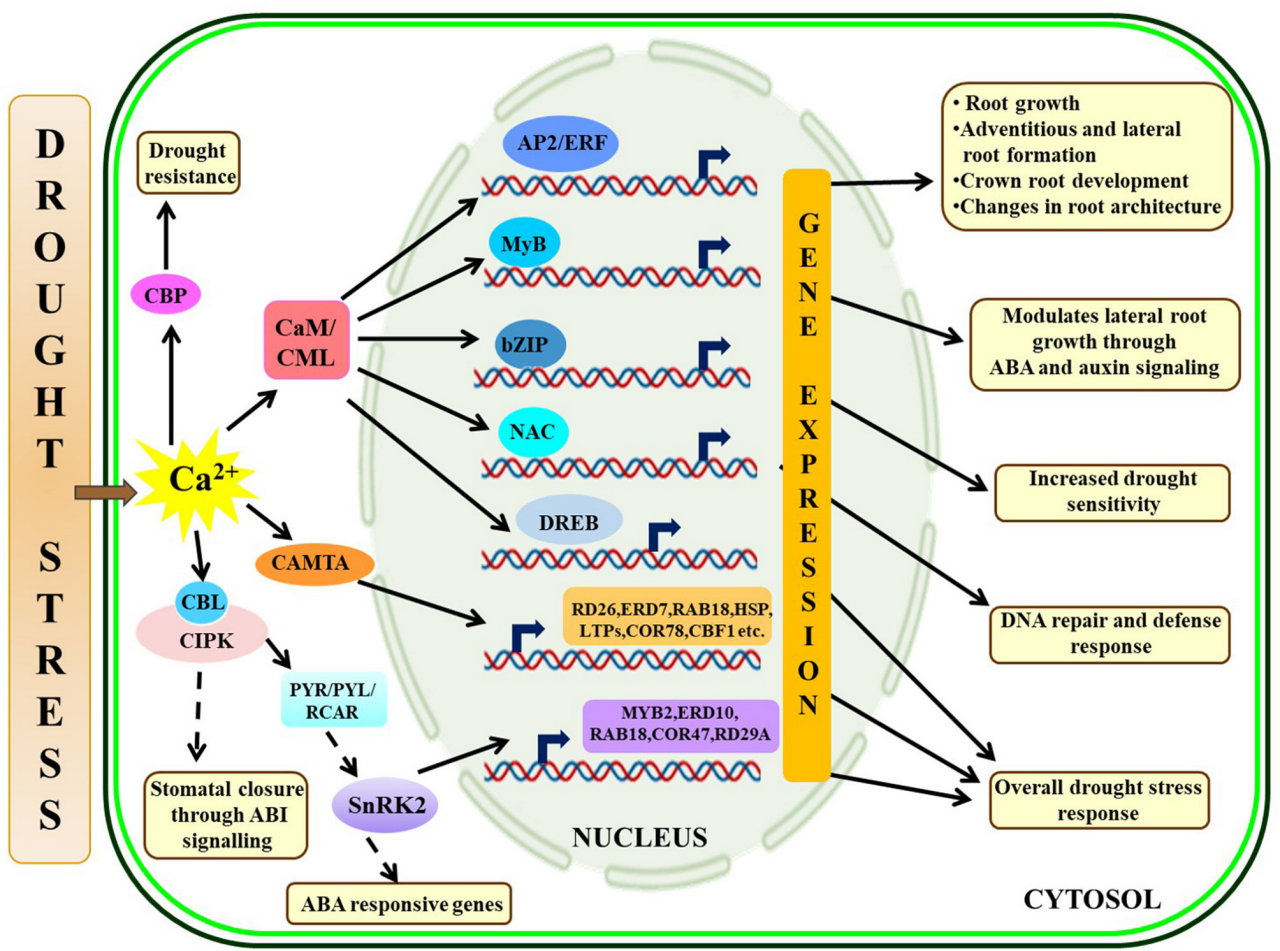
Illustrative representation of crosstalk among Ca^2+^ signaling components under drought-stressed condition, Drought stress-mediated [Ca^2+^]_cyt_ fluctuations activate Ca2+-interacting partners and provide response against the stress through varying mechanisms like CaM- and CAMTA-mediated gene expressions through several TFs conferring drought tolerance, CBL-CIPK interaction controls water loss through stomatal closure, PYR/PYL/RCAR regulates ABA pathway critical in stress-management. The solid arrow represents direct activation; the dotted arrow represents indirect activation via some intermediates. CaM, calmodulins; CAMTA, calmodulin-binding transcription activators; CIPK, CBL-interacting protein kinases.

Drought is one of the most challenging abiotic stresses that impart severe detrimental effects on growth and crop production. Plants encounter drought stress if the plant-available water is limited in the soil or when plants undergo higher transpiration losses compared to the root water uptake (Salehi-Lisar and Bakhshayeshan-Agdam, 2016). Roots are the first organs to sense drought stress and several sensory proteins, such as histidine kinases, receptor-like kinases, and G-protein-coupled receptors receive the external signal and amplify it through secondary messengers, such as Ca^2+^, reactive oxygen species (ROS), and IP3 (Jain et al., 2018). These secondary messengers will subsequently activate complex regulatory network controlling root growth under drought stress by regulating hormonal pathways and the expression of several TFs, such as APETALA 2/ethylene-responsive element binding factor (AP2/ERF), myeloblastosis viral oncogene homolog TF (MYB), basic leucine zipper (bZIP), and NAM/ATAF1/CUC2 (NAC) TF (Valliyodan and Nguyen, 2006; Jung et al., 2017). Similar to SOS signaling during salt stress, some CBL proteins (SOS3 homolog) interact with CIPK (SOS2 homolog) to confer abscisic acid (ABA) and drought stress signaling. An earlier study in *Arabidopsis* explains that a CBP SCaBP5 (CBL1) interacts with a protein kinase, PKS3 (CIPK15), to regulate ABA-dependent seed germination, stomatal opening, and subsequent gene expression (Guo et al., 2002). Additionally, PKS3 physically binds with ABI1 (ABA-insensitive 1) and ABI2, which are protein phosphatase 2C (PP2C) protein and are supposed to negatively regulate certain features of the ABA cascade, such as stomatal closure (Guo et al., 2002). In *Arabidopsis*, ABA-dependent stomatal closure is positively regulated by SnRK2 protein kinase (SRK2E/OST1/SnRK2.6) containing two C-terminal domains where domain I work in an ABA-independent manner and domain II is activated in presence of ABA (Yoshida et al., 2006). It has been found that SRK2E/OST1 physically interacts with ABI1 through domain II and is involved in ABA and osmotic stress signaling to control stomatal closure in *Arabidopsis*. Further studies identify that CBL9 interacts with CIPK3 and forms a module for ABA signaling and in addition to that abscisic acid repressor 1 (ABR1) acts as a downstream component of CIPK3 (Pandey et al., 2008; Sanyal et al., 2017). Another study in *Arabidopsis* identifies CML20 as a negative regulator of ABA-induced stomatal opening while the mutant *Arabidopsis* lines (cml20) show relatively less water loss from leaves and more drought tolerance (Wu et al., 2017). Moreover, drought and ABA stress upregulate transcript expressions of several stress-responsive genes, such as *MYB2, RAB18, ERD10, COR47*, and *RD29A* in the cml20 lines. On contrary, another CBP (CBP60g) acts as a positive regulator of drought stress in *Arabidopsis* (Wan et al., 2012). Classical model on ABA-mediated drought stress signaling depicts that upon binding with ABA, pyrabactin resistance 1/PYR1-Like/Regulatory components of ABA receptors (PYR/PYL/RCAR) inactivate PP2C which eventually activates SnRK2s, other ion channels, and TFs to turn on ABA-responsive genes (Hubbard et al., 2010). The mechanistic action of different environmental cues controlling the ABA signaling and other CBPs is nicely reviewed by Kumar et al. (2019). Hypo-methylation of CaM might be changing their binding partners and subsequent downstream signaling to depict salt and dehydration tolerance (Banerjee et al., 2013). Several CDPKs are also found to be involved in drought stress signaling (Xu et al., 2010; Ho et al., 2013; Pandey et al., 2013). Functional characterization of *OsCDPK1* in rice (*Oryza sativa*) reveals negative regulation of gibberellic acid biosynthesis while it activates a 14-3-3 protein (GF14c) and improves drought tolerance in transgenic seedlings during constitutive expression (Ho et al., 2013). Another study in *Arabidopsis* states that *CAMTA1* mutant (camta1) plants are highly susceptible to drought stress, and CAMTA1 further regulates the expression of several stress-responsive genes and TFs, such as RD26, ERD7, RAB18, LTPs, COR78, CBF1, heat shock proteins (HSPs), and AP2-EREBP (Pandey et al., 2013). An interesting study in maize (*Zea mays*) establishes that ZmCCaMK (calcium/CaMs-dependent protein kinase) phosphorylates a NAC TF (ZmNAC84) and induces antioxidant defense by activating downstream genes to enhance drought tolerance (Zhu et al., 2016). The overall Ca^2+^ signaling under drought stress is portrayed in Figure 2.

### Heat Stress

Calcium ion and CaM are important components in mediating the heat shock (HS) response in plants. Similar to other stresses, the increase in [Ca^2+^]_cyt_ is the first step in the HS signal transduction pathway. Transgenic tobacco (*Nicotiana tabacum*) plants expressing aequorin, a luminescent protein, exhibit an increase in [Ca^2+^]_cyt_ upon heat treatment. Additionally, exogenous application of Ca^2+^ results in improved thermo-tolerance, whereas the effect is diminished in the presence of Ca^2+^ chelators and inhibitors (Gong et al., 1998). Plants cells when exposed to high temperatures mobilize HSPs or molecular chaperones to aid in proper protein folding for mitigating the damage caused by heat stress. Laser confocal and fluorescence microscopic studies in wheat demonstrated that intracellular Ca^2+^ levels were increased within 1 min upon exposure to heat stress at 37°C. Furthermore, HS at 37°C upregulates *CaM 1-2* expression after 10 min, while two HSP genes, *hsp26* and *hsp70*, are induced later on. The expression of these HSP genes is upregulated after exogenous Ca^2+^ application, and their expressions are downregulated in the presence of CaM antagonists, Ca^2+^ chelators, and Ca^2+^ channel blockers (Liu et al., 2003). Similarly, maize seedlings pre-treated with CaCl_2_ showed enhanced thermotolerance coupled with a remarkable increase in CaM accumulation upon heat stress exposures (Zeng et al., 2015). The purified heat-inducible homolog of Hsp70 (DgHsp70) from orchard grass (*Dactylis glomerata*) possesses ATPase, holdase, and ATP-dependent foldase activity along with a CaM-binding domain (CaMBD). Moreover, binding to AtCaM2 in the presence of Ca^2+^ affects the ATPase and foldase activities of DgHsp70 protein (Cha et al., 2012). *AtCaM3* plays a pivotal role in heat stress signaling by regulating the activity of CaM-binding protein kinase (AtCBK3) which eventually controls HS TFs (AtHSFA1a) and HS protein genes through phosphorylation or dephosphorylation modulating heat stress response in *Arabidopsis* (Zhang et al., 2009). Furthermore, *AtCaM3* acts as a downstream factor in nitric oxide (NO) signaling resulting in the activation of heat shock factors (HSFs), accumulation of HSPs, and development of thermotolerance (Xuan et al., 2010). In a similar manner, Ca^2+^/CaM signaling is involved in HS response and an increase in the expression of Ca^2+^/HS-related proteins in rice (Wu et al., 2012). In *Arabidopsis*, AtPP7, a Ser/Thr phosphatase, is found to be induced under heat stress and known to interact with CaM in a Ca^2+^-dependent manner (Zeng et al., 2015). Moreover, AtPP7 is also found to interact with AtHSF1, an HS TF, thereby, suggesting its possible involvement in the regulation of the HSP genes (Liu et al., 2007). *Vitis amurensis* (*V. amurensis*) is a wild species of grapevine having high resistance to cold and disease. Overexpression of *VaCPK29* improves heat tolerance in *Arabidopsis* (Dubrovina et al., 2017). Another finding in *V. amurensis* depicts the differential regulations of various *VaCaM*s and *VaCML*s in response to an array of abiotic stresses, such as salt, cold, heat, and osmotic stress (Dubrovina et al., 2019). Altogether, 79 CML genes have been identified in the Chinese cabbage (*Brassica rapa* ssp. *pekinensis*), and expressions of many *BrCML*s, particularly *BrCML21–1*, are upregulated under heat stress (Nie et al., 2017). However, comprehensive research is warranted to elucidate the specific mechanisms by which these genes are regulated in different plants.

Heat stress causes a rise in intracellular levels of [Ca^2+^]_cyt_ which then combines with CaM and activates L-glutamate decarboxylase (GAD) for subsequent decarboxylation of glutamate (Glu) into γ-amino butyrate (GABA). However, calcium and CaM inhibitors block the GABA accumulation in *Arabidopsis* seedlings (Locy et al., 2000). CaM binding to GAD plays an important role in the normal development of plants while GABA is found to impart partial protection against heat stress by improving the leaf turgor, increasing the concentration of protective osmolytes, and reduction in the oxidative damage (Nayyar et al., 2014). Furthermore, transcriptomic analyses of rice reveal that *OsMSR2* (multi-stress-responsive gene 2), a novel CML is strongly upregulated under various abiotic stresses, such as cold, heat, and drought stress (Xu et al., 2011).

### Cold Stress

**Figure 3:**
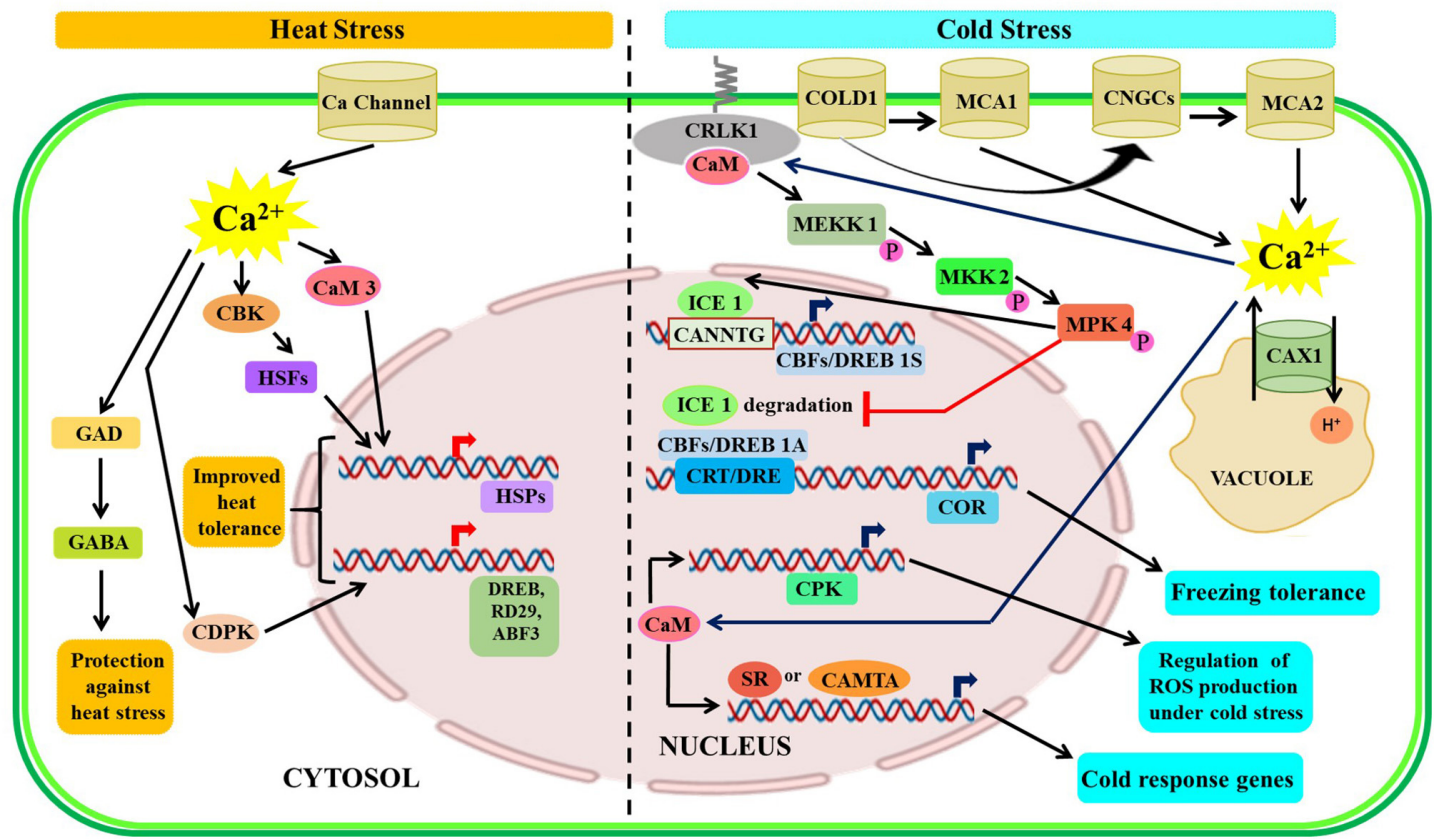
Illustrative representation of crosstalk among Ca^2+^ signaling components under temperature stressed condition. Upon sensing heat stress, plants respond through increased expression of several HSPs and other proteins like DREB, etc., along with activation of the GAD-GABA pathway. During cold stress, freezing tolerance is achieved through upregulated expression COR, ICE1, etc. CRLK, calcium/calmodulin-regulated receptor-like kinase 1; ICE1, inducer of CBF expression 1; CRT/DRE-C, repeat/dehydration-responsive element; SR, serine/arginine-rich; HSPs, heat shock protein; GAD, L-glutamate decarboxylase; GABA, γ-amino butyrate.

Plant responses to cold stress involve complex crosstalk through activation of several signaling pathways that could improve the chilling and freezing tolerance of plants. Cold temperature is perceived by various cold sensors localized in the plasma membrane which eventually led to a transient increase in the cytosolic or nuclear Ca^2+^ levels followed by activation of the downstream signaling cascade (Yuan et al., 2018). The rate of cooling also plays an important role in temperature sensing, a faster rate of cooling results in increased sensitivity along with increased Ca^2+^ response, whereas prolonged or repeated exposure to low temperatures attenuates the response (Knight and Knight, 2012). An earlier study revealed that CAX1 might have played a vital role in the cold stress response of *Arabidopsis* (Dodd et al., 2010). Additionally, two calcium-permeable mechano-sensitive channels have been detected as a regulator of cold-induced increment of cytosolic Ca^2+^ levels (Mori et al., 2018). In *Arabidopsis*, Yang et al. (2010) reported CRLK1 as a novel plasma membrane-localized calcium/CaM-regulated receptor-like kinase 1 containing two CaM-binding sites, which is stimulated in response to cold and oxidative stress and induces various cold-responsive (COR) genes, such as *CBF1, RD29A, COR15a*, and *KIN1*. Further research studies affirmed that an increase in cellular Ca^2+^ level caused CRLK1 to phosphorylate a specific mitogen-activated protein kinase kinase kinase (MEKK1), followed by the activation of protein kinase kinase 2 (MKK2) and MAPK signaling cascade leading to the activation of cold response genes (Furuya et al., 2013, 2014). Several Ca^2+^/CaM-binding proteins, such as *PsCCaMK* in pea (*Pisum sativum*), CaM-binding membrane transporter-like proteins (MCamb1 and MCamb2) in *Physcomitrella patens, OsCPK24* in rice and TCH, and a CaM-related protein, in *Arabidopsis*, show upregulation upon cold stress (Takezawa and Minami, 2004; Zeng et al., 2015; Liu et al., 2018). In common bean (*Phaseolus vulgaris*), the Ca^2+^-CaM-dependent NAD kinase (NADK) activity has increased after 60 min and 120 min of cold-shock (Ruiz et al., 2002). Further study speculates that CPKs induces a signaling cascade involving ROS, NO, and MPKs for ABA-dependent cold adaptation (Liu et al., 2018; Lv et al., 2018). The mechanistic action of CPKs under cold stress has been studied in a wide range of crops. The *ZmCPK1* in maize acts as a negative regulator of the cold stress response (Weckwerth et al., 2015); while *PeCPK10* in *Populus euphratica* (Chen et al., 2013), *OsCPK7, OsCPK13*, and *OsCPK17* in rice (Saijo et al., 2000; Komatsu et al., 2007; Almadanim et al., 2017), and *AtCPK1* in *Arabidopsis* act as positive regulator conferring cold tolerance (Almadanim et al., 2017). Furthermore, CaM3, an isoform of CaM, mediate reduced expression of the COR genes, such as *KIN1/2* and *LT178*, which affirmed that CaM3 exerted a negative effect on the COR expression probably by the activation of the Ca^2+^-ATPase (Townley and Knight, 2002). Finally, Ca^2+^-binding TF like CAMTA3 could bind to the promoter region of the *CBF2*/*DREB1c* gene and positively regulates their expression to confer freezing tolerance in plants (Doherty et al., 2009). The well-documented basic helix-loop-helix (bHLH) TF inducer of CBF expression 1 (ICE1) is the modulator of CBF genes during cold stress (Chinnusamy et al., 2003; Lindemose et al., 2013). When MYB15 and ICE1 interacted, they bound to the DREB1A/CBF3 promoter to regulate cold stress tolerance. Another plausible role of ICE1 is to act as a co-receptor for the cooling induction of Bon1-associated protein 1 (BAP1), which is responsive to cold stress (Zhu et al., 2011). The overall Ca^2+^ signaling under heat and cold stress is depicted in Figure 3.

### Heavy Metal/Metalloid Stress

Ever-increasing anthropogenic activities in recent years, such as industrialization and urbanization along with non-judicious use of inorganic fertilizer and pesticide containing heavy metals in agricultural fields, have led to severe heavy metal/metalloid pollution in soil and environment, which is malicious to the local ecosystem. The most common heavy metals/metalloid are lead (Pb), chromium (Cr), iron (Fe), arsenite (AsIII), arsenate (AsV), zinc (Zn), cadmium (Cd), copper (Cu), mercury (Hg), aluminum (Al), and nickel (Ni) (Wuana and Okieimen, 2011).

Lead ions (Pb^2+^) are capable of binding to the Ca-binding sites of CaM, eventually gaining entry into plant cells through Ca^2+^ permeable channels (Gorkhali et al., 2016). *Arabidopsis* CNGC*s* and tobacco *NtCBP4* are involved in the transport of Ca^2+^ ions during signal transduction and overexpressing *NtCBP4* confers Ni^+^ tolerance, but Pb^2+^ hypersensitivity (Moon et al., 2019). Interestingly, the expression of a truncated *NtCBP4* gene, which lacked a major part of the C-terminal half containing the cyclic nucleotide-binding domain and CaMBD in transgenic tobacco plants resulted in increased Pb^2+^ tolerance associated with reduced Pb^2+^ accumulation, while overexpression of full-length *NtCBP4* led to Pb hypersensitivity along with overaccumulation of Pb. In rice roots, Pb^2+^ treatment stimulates ROS production, Ca^2+^ accumulation, and activation of MAPKs in addition to cell death and stunted root growth. Conversely, pre-treatment of the roots with an antioxidant, CDPK-inhibitor (W7), and an nicotinamide adenine dinucleotide phosphate (NADPH) oxidase inhibitor effectively reduce the Pb^2+^-induced cell death and MAPK activity depicting the interplay between CDPK and ROS signaling under Pb^2+^ stress (Huang and Huang, 2008).

Cadmium is extremely phytotoxic even in low concentrations resulting in reduced photosynthetic rate, impeded stomatal conductance, and adverse effects on several biochemical and physiological activities in plants (Guo et al., 2019). Cd-stress depicted a detrimental effect in root growth of white clover (*Trifolium repens*) and *Arabidopsis* seedlings (Brunetti et al., 2011; Stravinskiene and Račaite, 2014). *Arabidopsis* root hair growth is also inhibited by Cd^2+^ as it affects the cytosolic Ca^2+^ gradient required for root growth (Fan et al., 2011). Cd^2+^ can also displace Ca^2+^ from CaM proteins, thus affecting calcium-dependent signaling (Baliardini et al., 2015). An increase in [Ca^2+^]_cyt_ levels occurs after Cd^2+^ exposure, which in turn activates the NADPH oxidase enzyme and transient H_2_O_2_ accumulation followed by superoxide (${\text{O}}_{2}^{-}$) accumulation in the mitochondria causing oxidative damage (Garnier et al., 2006). Baliardini et al. (2015) identified the probable role of the *CAX1* gene in Cd^2+^ tolerance. Several reports revealed that the application of Ca^2+^ played a crucial role in alleviating the damages caused by Cd^2+^ uptake in plants (Zorrig, 2012; Moon et al., 2019).

In a similar manner, pre-treatment with Ca^2+^ alone or in combination with other compounds can ameliorate the toxicity of Al, Cr, and Ni in different plants (Mozafari, 2013; Fang et al., 2014; Hossain et al., 2014). Ca^2+^ oscillation in rice roots was monitored using a Ca^2+^ indicator, and ROS-specific dyes revealed a significant increase in Ca^2+^ after exposure to arsenate [As(V)]. Further, transcriptome analysis of rice roots upon As(V) exposure reveals overexpression of various calcium-related proteins and kinases, such as CaMs, CBLs, CDPKs, and CIPKs, coupled with several TFs, MAPKs, NADPH oxidases along with genes involved in abiotic stresses (Huang et al., 2012). Additionally, transcript analyses depict upregulation of different Ca^2+^/CaM-binding partners, such as CDPKs in rice upon Cr^6+^ stress, CaM in basidiomycetous yeast species, *Cryptococcus humicola*, consequent to Al stress, and another CaM in zucchini (*Cucurbita pepo*) upon Ni stress (Huang et al., 2014; Zhang et al., 2016; Valivand et al., 2019). Transcriptomic analysis of Tibetan Plateau annual wild barley (*Hordeum spontaneum)* affirms that multiple CIPKs are playing a pivotal role to mitigate heavy metal toxicity (Hg, Cd, Cr, Pb, and Cu) and other climatic vagaries such as salt, drought, ABA, cold, and heat stress (Pan et al., 2018). Lanthanum (La^III^), a rare earth element, is reported to trigger an increase in Ca^2+^ levels in the cell, besides interacting with CaM, consequently affecting the expression and conformation of CaM (Wang et al., 2016; Zhang et al., 2016). At low concentrations, La^III^ could bind with the two Ca-binding sites of the extracellular CaM by electrostatic attraction to improve CaM function and *vice versa* (Wang et al., 2016).

## Ca^2+^ Signaling in Plant-Biotic Factor Interactions

The ability of plants to discriminate between harmful biotic agents (pathogens and pests) and beneficial symbiont subsequently exerting differential response either by limiting invasion or by promoting association is the key to survival.

### Symbiosis

Most of our current understanding of symbiosis signaling is based on the work done mainly on the association of rhizobia and mycorrhiza with *Lotus japonicus* and *Medicago truncatula*. Besides rhizobia and mycorrhiza, beneficial interactions also occur with endophytic fungi and plant-growth-promoting bacteria which colonize at the root of the host and result in better plant performance (Khare et al., 2018). Symbiotic interactions start through sequential cytoplasmic and nuclear Ca^2+^ elevations in the plant cell, and the responses depend on the amplitude, duration, frequency, and location of Ca^2+^ signature and selective activation of specific calcium channels in cellular membranes (Whalley and Knight, 2013; Yuan et al., 2017).

Plant root-secreted flavonoids and strigolactones are sensed by *Rhizobium sp*. and mycorrhizal fungi, respectively. Upon recognizing these compounds, symbionts produce modified lipo-chitooligosaccharides, such as nodulation (Nod) factors in the case of rhizobial symbiosis, and mycorrhizal (Myc) factors in the case of arbuscular mycorrhizal fungi symbiosis (Oldroyd, 2013). Upon recognition and binding with the Nod factor, LysM receptor-like kinases form heterocomplex on the cell membrane and activate the cytosolic kinase domain of LysM, triggering cytosolic Ca^2+^-oscillation within seconds to minutes. Nuclear Ca^2+^ oscillation is generated through some yet unidentified secondary messenger activated by LysM receptor-like kinases (Oldroyd, 2013). Successful formation of both root nodule and arbuscular mycorrhizal symbiosis requires activation of a common symbiotic pathway composed of eight components, SYMRK (in *L. japonicus*)/DMI2 (in *M. truncatula*), POLLUX/DMI1, CASTOR, NENA, NUP85, NUP133, CCaMK, and CYCLOPS/IPD3 (Singh and Parniske, 2012; Oldroyd, 2013). Nuclear membrane-localized Ca^2+^-regulated cation channels, such as CASTOR and POLLUX in *L. japonicus* (Charpentier et al., 2008) and DMI1 in *M. truncatula* (Ané et al., 2004), have been identified as crucial for symbiotic Ca^2+^ oscillations in the root cell nucleus (Singh et al., 2014). Besides these, three components of the nuclear pore complex, i.e., NUP133, NUP85, and NENA, are also essential for symbiotic signaling (Binder and Parniske, 2014). Nucleus-localized CCaMK, calcium- and CaM-dependent serine/threonine protein kinase, directly interacts with CYCLOPS and phosphorylates it, which is critical for decoding the calcium oscillations (Singh et al., 2014). The mechanism by which CCaMK-CYCLOPS activates specific downstream signaling for nodulation or mycorrhiza formation yet remains unresolved.

Transcription factors, such as nodulation signaling pathway 1 (NSP1) and NSP2, are critical for rhizobium-specific gene expression as it interacts with the promoters of rhizobium-induced genes, such as *nodule inception* (*NIN*) and *ERN1* (Cerri et al., 2012). Both the CCaMK–CYCLOPS complex and the NSP1–NSP2 complex are vital for driving specific genes expression associated with successful nodule formation. In the case of mycorrhizal signaling, expression of mycorrhiza-specific *RAM2* (required for arbuscular mycorrhization 2) gene is controlled by the same signaling complexes with a substitution of NSP1 by RAM1 (Gobbato et al., 2013).

### Pathogenesis

Plant's first line of defense is activated by interaction either between plasma membrane bound pattern-recognition receptors (PRRs) with several elicitors, such as pathogen-associated molecular patterns (PAMPs), microbe-associated molecular patterns (MAMPs), endogenous damage-associated molecular patterns (DAMPs), or compounds produced from cell damage due to pathogenic activity (Dodds and Rathjen, 2010). PRR initiates an overall defense response through activating PAMP-triggered immunity (PTI), followed by rapid activation of MAPKs, oxidative burst, and Ca^2+^ influx at the plasma membrane (Ranf et al., 2011) and finally augmenting biosynthesis of plant defense hormones, namely, salicylic acid (SA), jasmonic acid (JA), and their derivatives (Vlot et al., 2009). Microbe-induced transient increase of Ca^2+^ flux is the most important upstream signaling event leading to SA biosynthesis (Du et al., 2009). CaM-binding CBP60 family TFs, CBP60g, and systemic-acquired resistance deficient 1 (SARD1) act as a positive regulator of isochorismate synthase 1 (ICS1) expression, a key enzyme in the SA biosynthesis necessary for inducing systemic-acquired resistance (SAR) (Zhang et al., 2010). The importance of CaM binding of CBP60g and other related proteins (CBP60a) in defense signaling has been well-studied in *Arabidopsis* (Wang et al., 2009; Truman et al., 2013). The crucial role of CAMTA as an early integration node of both PTI and ETI signaling pathways came from a transcriptomics study by comparative analysis of different CAMTA conditional mutant transgenic lines of *Arabidopsis* (Jacob et al., 2018). CAMTA3 negatively regulates expression of enhanced disease susceptibility 1 (EDS1) and brassinosteroid insensitive 1-associated kinase 1 (BAK1) interfering with SA and JA signaling, respectively (Du et al., 2009; Rahman et al., 2016). Plants with CAMTA3 mutation exhibit greater resistance to pathogenic bacteria (*Pseudomonas syringae, Botrytis cinereal*, and *Sclerotinia sclerotiorum*) but become more susceptible to insect attack (*Trichoplusia ni*) (Galon et al., 2008; Rahman et al., 2016). Similarly, AtCAMTA3 negatively regulates plant immunity by conferring non-host resistance to *Xanthomonas oryzae* (Rahman et al., 2016).

The first evidence linking CMLs with plant defense came from overexpression of soybean (*Glycine max*) *CML1* and *CML2* (previously, SCaM-4 and SCaM-5) in tobacco, *Arabidopsis*, and soybean causing increased resistivity against a wide range of virulent and avirulent pathogens (Heo Do et al., 1999; Park et al., 2004; Rao et al., 2014). Validation from autologous and heterologous systems highlights the role of CaM/CMLs in plant immune responses through modulating the hypersensitive response (HR) (Chiasson et al., 2005; Ma et al., 2008). *Arabidopsis* CML8 and CML9 are also acting as positive regulators of defense mechanisms against different strains of *P. syringae* through activation of SA-dependent defense responses (Zhu et al., 2017). Additionally, CML9 interacts with a plant-specific TF, pseudo-response regulator 2 (PRR2) in planta which is a player in SA-dependent immunity response in the plant (Cheval et al., 2017). Furthermore, CML37 and CML42 play contrasting roles in insect herbivory-induced resistance in positive and negative manners, respectively (Vadassery et al., 2012; Scholz et al., 2014).

**Figure 4:**
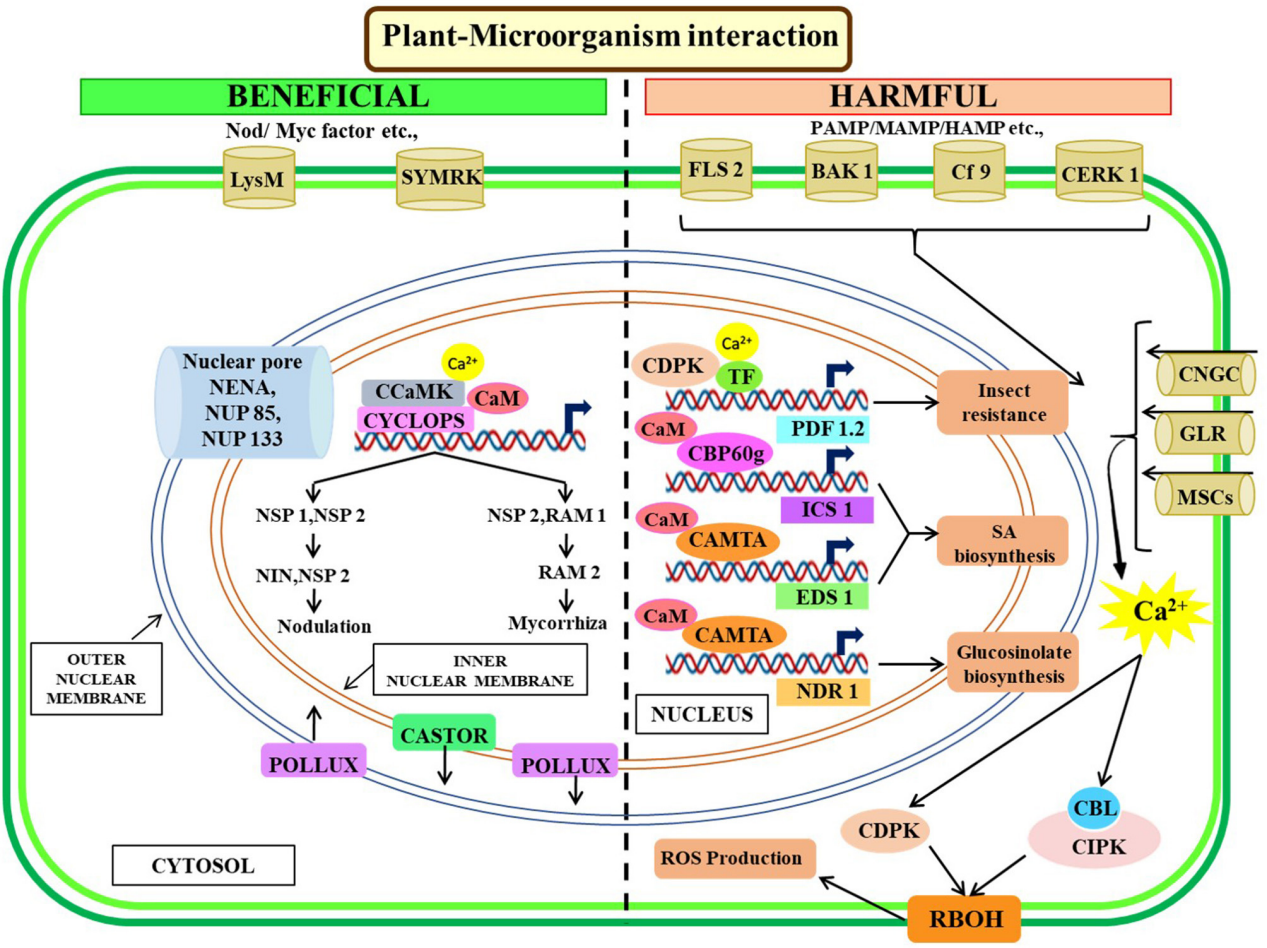
Illustrative representation of crosstalk among Ca^2+^ signaling components for plant's biotic interactions. Symbiosis signaling starts with the perception of Nod/Myc factors and subsequent Ca^2+^ signaling mediated activation of either nodulation through the expression of NIN, NSP1, NSP2, or mycorrhiza through RAM1, RAM2 expression. Nuclear membrane localized calcium channels (POLLUX, CASTOR) and nuclear pore complex are essential for plant-microorganism symbiosis. PAMP/MAMP/HAMP activates resistance pathways agents, biotic agents, through SA, glucosinolate biosynthesis, and ROS production during pathogenesis. The membrane-localized transporters trigger Ca^2+^ in the cytoplasm upon interaction with harmful biotic agents which eventually triggers the CBL-CIPK-CAMTA-mediated signaling cascade for downstream gene expression. NDR, non-race-specific disease resistance1; MSC, mechanosensitive ion channels; SYMRK, symbiosis receptor-like kinase; FLS2, flagellin sensing 2; CERK1, chitin elicitor receptor kinase 1; CAMTA, calmodulin-binding transcription activators; CIPK, CBL-interacting protein kinases; CBLs, calcineurin B-like proteins.

Advances in functional genomics in recent times shed light on the importance of CDPKs in plant immune responses. As a family, CDPKs exert both positive and negative regulations on the immune response. Several members of *Arabidopsis* CDPKs, i.e., CPK4, 5, 6, and 11 are transiently activated within a few minutes after recognition of common 22-amino-acid long bacterial flagellin epitope flg22 (Boudsocq et al., 2010). These CPKs along with MAPKs positively regulate innate immune signaling perceived by PAMP receptor FLS2 (Boudsocq et al., 2010). *In vitro* phosphorylation studies revealed that CPKs can also phosphorylate various WRKY TF-controlling downstream immunity-related crucial gene expressions (Boudsocq et al., 2010; Gao et al., 2013). *Arabidopsis* mutant screening identified two CDPKs (AtCPK3 and AtCPK13) as important transcriptional regulators of plant defensing gene *PDF1.2*. HSF HsfB2a promotes transcriptional activation of *PDF1.2*, after being phosphorylated by CPK3-or CPK13 in response to wounding by herbivores, such as *Spodoptera littoralis* (Kanchiswamy et al., 2010). Pathogen-induced Ca^2+^ elevations in cytosol activate potato CDPKs (StCPK4 and StCPK5) which in turn stimulates respiratory burst oxidase homolog protein B (RBOHB) (Kobayashi et al., 2007). RBOH gene family encodes integral membrane protein NADPH oxidases, which are a key component of innate immunity due to its central role in ROS production and subsequent oxidative burst and CPK5-mediated *in vivo* phosphorylation of RBOH homolog D in *Arabidopsis* (Dubiella et al., 2013). Moreover, CDPKs control cell death in tobacco after recognizing *Cladosporium fulvum* through interaction between pathogen race-dependent elicitor Avr4/Avr9 and disease-resistant gene Cf9 (Romeis et al., 2001). In the *japonica* rice cultivar Nipponbare, overexpression of CDPK enhanced blast resistance by preventing *Magnaporthe oryzae* penetration (Bundó and Coca, 2016). In addition to enhancing plant resistance, CDPKs also perform a negative role in plant defense (Freymark et al., 2007; Asano et al., 2012; Monaghan et al., 2014). However, the function of CDPKs remains unresolved due to duplicity and functional redundancy in higher plants which further warrants comprehensive research to uncover the role of this protein toward plant defense mechanisms. The intricate Ca^2+^ signaling pathways toward regulating plant-biotic factors interaction are illustrated in Figure 4.

## Plant Homeostasis Under Multiple Stresses

Plants with complex systems and sessile nature have to face multiple stresses when the growing environment is suboptimal and ultimately hinders the expression of complex traits, such as yield and other developmental activities. On the verge of climatic aberrations, plants are becoming more vulnerable toward biotic and abiotic impulses which further elicit changes in the physiological and molecular levels within the plant system to cope up with the vagaries without compensating yield. As plant responses toward multiple stresses are mostly additive in nature, the understanding of the crosstalk between multiple signaling components is the need of the hour to ameliorate the issue in a pragmatic way. The involvements of Ca^2+/^CaM-related multiple pathways, sensory components, regulatory proteins, and TFs have a plausible role in plant defense mechanisms against biotic and abiotic cues to maintain plant homoeostasis.

**Figure 5:**
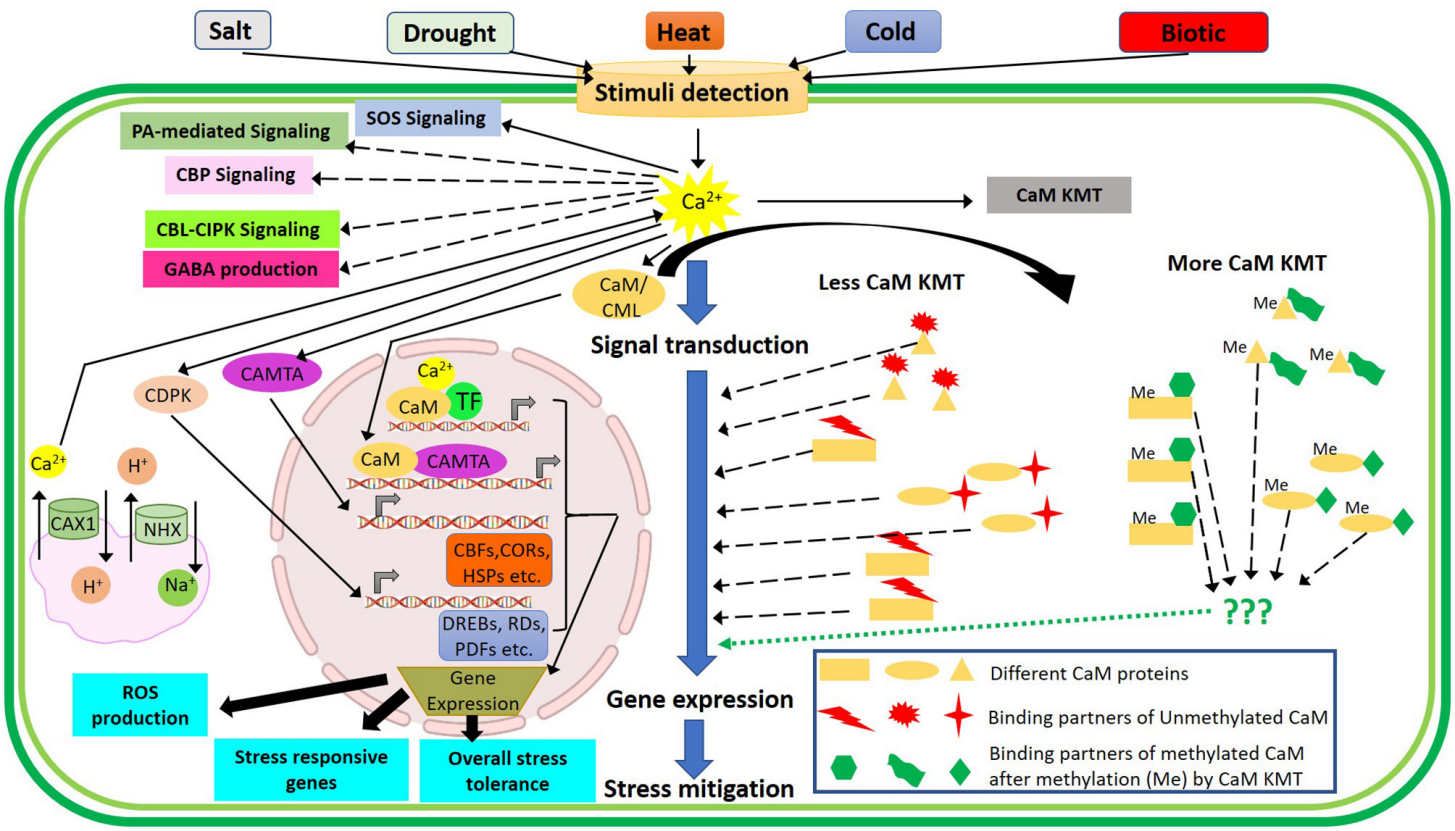
Illustrative representation of crosstalk among Ca^2+^ signaling components associated with multiple stimuli perceived by the plants. Spike in [Ca^2+^]_cyt_ triggers several pathways like SOS signaling, PA-mediated signaling, CBP signaling, CBL-CIPK signaling, GABA production, CAX1, and NHX regulation and activates CDPK, CAMTA, CaM, and CML for subsequent expression of stress-responsive genes in the nucleus depending on the types and magnitudes of stimuli. Calmodulin-lysine N-methyltransferase (CaM KMT) methylates CaM proteins in the cytosol while the methylated CaM (Me) and unmethylated CaM depicts differential interactions with CaM-binding partners (depicted in green and red shapes, respectively) and subsequently regulate the downstream signaling components to mitigate various stresses. CaM, calmodulin; CIPK, CBL-interacting protein kinases; CBLs, calcineurin B-like proteins; CDPKs, Ca^2+^-dependent protein kinases; CML, calmodulin-like proteins.

For plant growth and development, nitrate (${\text{NO}}_{3}^{-}$) is one of the vital forms of inorganic nitrogen uptake by crop plants. The plethora of ${\text{NO}}_{3}^{-}$ sensors has been documented in *Arabidopsis*. Among these, NRT1.1 is well-studied and presumed to act upstream of several signaling pathways (Sun and Zheng, 2015). Typically, NRT1.1 is the vital component of nitrate peptide transporter family (NPF) related to membrane transporters (Léran et al., 2014) and governs a range of responses related to primary ${\text{NO}}_{3}^{-}$ response coupled with germination, flower initiation, development of root and shoot growth, and tolerance or sensitivity to various ${\text{NO}}_{3}^{-}$-dependent abiotic cues *viz*., temperature extremities, moisture stress, salinity, ${\text{NH}}_{4}^{+}$, and Cd toxicities (Mao et al., 2014; O'Brien et al., 2016; Álvarez-Aragón and Rodríguez-Navarro, 2017; Jian et al., 2018; Teng et al., 2019). Therefore, NRT1.1 has been recognized as a “transceptor” that contemplates the dual role of transporter and receptor related to ${\text{NO}}_{3}^{-}$ transport and sensing function (Ho et al., 2009; Gojon et al., 2011). Interestingly, NRT1.1 has a dual affinity of ${\text{NO}}_{3}^{-}$ and acts as both low-affinity and high-affinity transport systems (Wang et al., 2012). During this biphasic control of ${\text{NO}}_{3}^{-}$ signaling and transport, NRT1.1 forms a dimer by inducing/through crosstalk with CBL9-activated kinase, CIPK23 (Sun and Zheng, 2015; Wen and Kaiser, 2018). Studies have revealed that NRT1.1 ameliorated drought and salinity stress in both ABA-dependent and independent manners through modulating the activity of CIPK23. Furthermore, it has been found that CIPK23 interacts and phosphorylates the NRT1.1 along with ammonium transporter AMT1 toward triggering their activity to maneuver the ammonium toxicity (Ho et al., 2009; Straub et al., 2017; Tian et al., 2021). In addition to the crosstalk between the NRT1.1 sensor and Ca^2+/^CaM signaling components, Calmodulin-lysine N-methyltransferase (CaM KMT) has been identified as an important modulator of several signal transductions (Banerjee et al., 2013). CaM KMT trimethylates CaM at a specific lysyl residue and the methylation status of CaM plays an important role in the determination of CaM-binding partners in presence of Ca^2+^ (Magnani et al., 2010, 2012). The earlier report depicted that undermethylation of CaM in CaM KMT knockout mutant plants made it relatively more tolerant to salt, drought, and thermal stresses compared to CaM KMT overexpressed and control *Arabidopsis* plants through possible changes in CaM-binding partners (Banerjee et al., 2013). Similarly, another major regulatory TF, CAMTA, is involved in the regulation of multiple stress-related genes upon exposure to drought, temperature extremities, and biotic factors (Doherty et al., 2009; Du et al., 2009; Pandey et al., 2013). The overall Ca^2+^/CaM-mediated signaling upon exposure to multiple stimuli is depicted in Figure 5.

## Biotechnological Interventions

Unprecedented progress in modern molecular biology coupled with novel “OMICS” tools has enabled researchers to unravel the physiological and molecular mechanisms to combat stress through direct and unbiased monitoring of the complex interplay among stress signaling and response by plants. Modulating the gene activity through various biotechnological approaches, such as overexpression strategies, RNA interference-mediated gene silencing, or genome-editing technologies, has hastened crop improvement since the last three decades. Biotechnological interventions on various Ca^2+^/CaM-binding proteins viz., CaM, CML, CBP, CDPK, TFs, and others had resulted in the generation of stress-tolerant lines to mitigate the adverse effects of various abiotic and biotic stresses (Table 1). Although the specificity of an individual Ca^2+^/CaM-binding partner toward specific stress is obscure, out of the several Ca^2+^/CaM-binding proteins, CDPK/CIPK regulations were reported with several abiotic or biotic factors possibly due to their action in the upstream part of the signaling cascade (Pan et al., 2018). CAMTAs, being a TF also regulate several downstream genes associated with signaling cascade to regulate several stressors (Doherty et al., 2009; Rahman et al., 2016). Furthermore, another master regulator of Ca^2+^/CaM signaling is CaM KMT, and expressional regulation of CaM KMT in *Arabidopsis* depicts the involvement of CaM KMT in salt, drought, and cold stress signaling (Banerjee et al., 2013). Hence, the identification of master switch is of utmost priority to the scientific community to cope up with the inevitable abiotic and biotic stressors.

**Table 1:** Functional genomics studies on Ca^2+^/CaM-signaling components for conferring stress tolerance in plants.

**Signaling components**	**Gene**	**Plant**	**Genetic intervention**	**Type of stress**	**References**
CAM	AtCaM7	*Arabidopsis*	Overexpression	Light	Kushwaha et al., 2008
	CaM3	*Arabidopsis*	Overexpression	Cold	Townley and Knight, 2002
	OsCaM1-1	*Arabidopsis*	Overexpression	Heat	Wu et al., 2012
	SCaM-4, SCaM-4-5	Soybean, tobacco	Overexpression	Biotic	Heo Do et al., 1999; Rao et al., 2014
	HvCaM1	Barley	Overexpression and RNA interference	Salt	Shen et al., 2020
Kinases	VaCPK9	*Arabidopsis*	Overexpression	Heat	Dubrovina et al., 2017
	OsCDPK1	Rice	Overexpression	Drought	Ho et al., 2013
	OsCPK12	Rice	Overexpression	Biotic	Asano et al., 2012
	OsCPK24	Rice	Overexpression, knockdown mutation	Cold	Liu et al., 2018
	OsCPK4	Rice	Overexpression	Biotic	Bundó and Coca, 2016
	MdCPK1a	Tobacco	Overexpression	Salt and cold	Dong et al., 2020
	MKK2	*Arabidopsis*	Overexpression	Salt	Teige et al., 2004
	HsCIPK	Rice	Overexpression	Heavy metal, drought	Pan et al., 2018
	AtCPK6	*Arabidopsis*	Overexpression	Salt, drought	Xu et al., 2010
CMLs	CML24	*Arabidopsis*	Knockout mutation	Biotic	Ma et al., 2008
	CML8	*Arabidopsis*	Overexpression	Biotic	Zhu et al., 2017
CBPs	NtCBP4	Tobacco	Overexpression	Heavy metal	Moon et al., 2019
	NtCBP4	Tobacco	Truncated expression	Heavy metal	Sunkar et al., 2000
Transcription factors	SNAC1	Rice	Overexpression	Drought	Hu et al., 2006
	OsNAC14	Rice	Overexpression	Drought	Shim et al., 2018
	OsERF48	Rice	Overexpression	Drought	Jung et al., 2017
	OsbZIP52	Rice	Overexpression	Drought	Liu et al., 2012
	MYB96	*Arabidopsis*	T-DNA insertional mutation, overexpression	Drought	Seo et al., 2009
	DREB2A	*Arabidopsis*	Overexpression	Drought	Sakuma et al., 2006
Others	APR134	Tomato	Gene silencing	Biotic	Chiasson et al., 2005
	AtPP7	*Arabidopsis*	Knockout mutation, overexpression	Heat	Liu et al., 2007
	CaM KMT	*Arabidopsis*	Knockout mutation	Heat and cold	Banerjee et al., 2013
	SlSOS2	Tomato	Overexpression	Salt	Huertas et al., 2012

*CaM, calmodulins, CML, calmodulin-like proteins; CBP, calcium-binding protein*.

## Future Perspective

The agriculture sector is facing an unnerving challenge due to rapid climate change. In crop breeding programs, researchers are more focused on developing improved stress resilient varieties for better sustenance of mankind. Notable progress has been made in recent decades in the field of both basic and applied research studies highlighting the role of Ca^2+^ signaling as the core mechanisms and processes underlying abiotic and biotic stress adaptation in crop plants and decoding Ca^2+^ signature by employing various Ca^2+^-binding proteins viz., CaM, CML, CDPK, and others (Du et al., 2009; Hashimoto and Kudla, 2011; Banerjee et al., 2013; Meena et al., 2019) through intended genetic modifications. Functional genomics and mutation study in *Arabidopsis* deciphered the biological significance of several Ca^2+/^CaM signaling components associated with abiotic and biotic signal transduction processes but the picture is unclear in most of the food crops. As there is a huge redundancy for CaMs, CMLs, CAMTAs, and CDPKs in the plant genome, neither siRNA-mediated nor artificial microRNA (amiRNA)-mediated gene-silencing strategies would be sufficient to knockdown the effect of respective isoforms. In spite of overexpressing a single Ca^2+^/CaM-player to mitigate stress, gene stacking or gene pyramiding of multiple positive alleles would be more relevant to cope up with the situation. Furthermore, simultaneous editing (either gene knockdown or overexpression or both of several pathway components associated with particular stress) of multiple genes could alleviate certain stresses which would eventually improve crop fitness. Although stress is a complex phenomenon, and multiple stresses are encountered by the plant at the same time, Ca^2+^/CaM signaling pathways are ubiquitous in every stress signaling. Hence, overexpression and genome editing might be the most suitable approach to regulate the master regulator (*CaM KMT* or *CAMTAs*) for stress mitigation in the climate-resilient condition to fetch better yield in agricultural crops.

## Author Contributions

NP, AD, and JB conceptualized the review. NP and SH prepared the draft manuscript. AD, MM, and JB reviewed and edited the manuscript. HG made the figure after revision and corrected the manuscript. All authors contributed to the article and approved the submitted version.

## Funding

This publication was supported by SERB, DST, Govt. of India (File no. ECR/2018/000328) and Indian Institute of Technology Kharagpur as well as SRIC, IIT Kharagpur.

## Conflict of Interest

The authors declare that the research was conducted in the absence of any commercial or financial relationships that could be construed as a potential conflict of interest.

## Publisher's Note

All claims expressed in this article are solely those of the authors and do not necessarily represent those of their affiliated organizations, or those of the publisher, the editors and the reviewers. Any product that may be evaluated in this article, or claim that may be made by its manufacturer, is not guaranteed or endorsed by the publisher.
